# Revisiting the Factor Structure of the German WISC-V for Clinical Interpretability: An Exploratory and Confirmatory Approach on the 10 Primary Subtests

**DOI:** 10.3389/fpsyg.2021.710929

**Published:** 2021-09-14

**Authors:** Franz Pauls, Monika Daseking

**Affiliations:** ^1^Department of Clinical Psychology, Helmut-Schmidt-University/University of the Federal Armed Forces, Hamburg, Germany; ^2^Department of Educational Psychology, Helmut-Schmidt-University/University of the Federal Armed Forces, Hamburg, Germany

**Keywords:** intelligence assessment, structural validity, exploratory factor analysis, confirmatory factor analysis, clinical interpretability, German Wechsler Intelligence Scale for Children - Fifth Edition

## Abstract

With the exception of a recently published study and the analyses provided in the test manual, structural validity is mostly uninvestigated for the German version of the Wechsler Intelligence Scale for Children - Fifth Edition (WISC-V). Therefore, the main aim of the present study was to examine the latent structure of the 10 WISC-V primary subtests on a bifurcated extended population-representative German standardization sample (*N*=1,646) by conducting both exploratory (EFA; *n*=823) and confirmatory (CFA; *n*=823) factor analyses on the original data. Since no more than one salient subtest loading could be found on the *Fluid Reasoning* (FR) factor in EFA, results indicated a four-factor rather than a five-factor model solution when the extraction of more than two suggested factors was forced. Likewise, a bifactor model with four group factors was found to be slightly superior in CFA. Variance estimation from both EFA and CFA revealed that the general factor dominantly accounted for most of the subtest variance and construct reliability estimates further supported interpretability of the *Full Scale Intelligence Quotient* (FSIQ). In both EFA and CFA, most group factors explained rather small proportions of common subtest variance and produced low construct replicability estimates, suggesting that the WISC-V primary indexes were of lower interpretive value and should be evaluated with caution. Clinical interpretation should thus be primarily based on the FSIQ and include a comprehensive analysis of the cognitive profile derived from the WISC-V primary indexes rather than analyses of each single primary index.

## Introduction

The Wechsler scales are among the most frequently used diagnostic instruments worldwide for assessing a variety of cognitive abilities across different age groups. Following a long tradition, the Wechsler Intelligence Scale for Children - Fifth Edition. (WISC-V; Wechsler, 2014a) is a complex test battery for measuring intellectual performances of children and adolescents aged between 6 and 16years. Its conceptualization and development were mainly inspired by the theoretical ideas of Cattell, Horn, and Carroll, often referred to as the Cattell-Horn-Carroll model (McGrew, 2009; Schneider and McGrew, 2012). Representing a major revision of the WISC-IV (Wechsler, 2003), the WISC-V incorporates several significant changes. As one major modification, the WISC-V now redefines the four factors of the WISC-IV into a new second-order five-factor structure including the primary indexes *Verbal Comprehension Index* (VCI), *Visual Spatial Index* (VSI), *Fluid Reasoning Index* (FRI), *Working Memory Index* (WMI), and *Processing Speed Index* (PSI). Each of these indexes is derived using two out of 10 primary subtests. Moreover, VSI and FRI replaced the former WISC-IV primary index *Perceptual Reasoning Index* (PRI) as part of the new second-order five-factor structure of the WISC-V. This conceptual framework finally describes the *Full Scale Intelligence Quotient* (FSIQ) as an estimate of the general intellectual ability that is composed of five cognitive subdomains.

In order to examine the validity of the factor structure on an empirical basis, the test publishers conducted confirmatory factor analyses (CFA) and compared alternative factor structures to identify a theoretically sound model that accounted for the standardization data. CFA results provided in the test manual indicate that most of the five-factor model solutions were superior in terms of their model fit when compared to four-factor model solutions. Based on goodness-of-fit statistics, the second-order five-factor model was preferred and selected by the test publishers to best represent the WISC-V test structure (see Wechsler, 2014b, for a detailed description). Despite some attempts to support validity of the five-factor structure both in normative (e.g., Keith et al., 2006) and clinical samples (e.g., Weiss et al., 2013), however, there is still an ongoing debate on whether the WISC-V subtest performances are best described by a four- or a five-factor structure (Dombrowski et al., 2018). Recent studies indicated that the WISC-V factor structure might be best represented by four first-order factors (Canivez et al., 2016), whereas a five-factor structure could neither be satisfyingly replicated for the Canadian (Watkins et al., 2017), the French (Lecerf and Canivez, 2018), nor the Spanish WISC-V (Fenollar-Cortés and Watkins, 2018). In a recent report, McGill et al. (2020) have highlighted some methodological limitations related to several WISC-V standardization procedures.

In the light of these findings, the five-factor structure of the WISC-V has been called into question by some researchers (e.g., Beaujean, 2016; Canivez et al., 2018). Since shortly after the WISC-V was first published in the United States, the structural validation procedures reported in the test manual (Wechsler, 2014b) have been criticized as problematic (Beaujean, 2016; Canivez et al., 2017; Dombrowski et al., 2018). Besides failures in fully disclosing details about the CFA methods being used and the retention of FR as a redundant factor, one of the most frequently mentioned issues referred to failures in considering rival bifactor models when comparing alternative model solutions (Beaujean, 2015). Rather than specifying a higher-order factor as a superordinate general factor (*g*) that is only associated with and fully mediated by the lower-order factors, bifactor models describe *g* and the group factors at the same level of inference, featuring independent associations with the subtest indicators (Reise, 2012; Canivez, 2016). Another point of criticism relates to the fact that neither information about the sources of decomposed variances nor adequate estimates of model-based reliability are provided in the WISC-V test manual. However, estimating the proportions of decomposed variance can be crucial for determining how much interpretative emphasis one should place upon the factors included in the model under examination.

Since no results of exploratory factor analyses (EFA) are reported in the WISC-V test manual, these have additionally been conducted either on the overall standardization sample (Dombrowski et al., 2015; Canivez et al., 2016) or on specific age groups (Dombrowski et al., 2018). The according studies found support for four rather than five first-order factors, thus resembling the former WISC-IV factor structure. Comparable results have also been reported in studies conducting hierarchical or bifactor EFA and CFA on the Canadian (Watkins et al., 2017), French (Lecerf and Canivez, 2018), United Kingdom (Canivez et al., 2018), or Spanish versions of the WISC-V (Fenollar-Cortés and Watkins, 2018). Moreover, it could be shown that substantial proportions of subtest variance were due to *g*, whereas the group factors accounted for considerably smaller proportions of variance (Dombrowski et al., 2018). It was concluded that the FSIQ might be sufficiently well measured by the WISC-V subtests, whereas model-based reliability might be too weak for the WISC-V primary indexes to be meaningfully interpretable (Canivez et al., 2016; Rodriguez et al., 2016).

Even though analyses of the United States, Canadian, French, United Kingdom, and Spanish standardization data have already focused on the structural validity of the WISC-V, evidence is still needed to clarify which factor structure could best represent the German adaptation of the WISC-V. Apart from collecting and analyzing data from the German population, further significant changes were implemented as compared with the United States version of the WISC-V. These changes included the translation and adaptation of the entire set of verbal items, the exclusion of some complementary scores, and several modifications to verbal contents (see Wechsler, 2017a, for a detailed description). In a recently published study, Canivez et al. (2021) were the first to analyze the structural validity of the 15 WISC-V primary and secondary subtests based on the correlation matrix of the German standardization sample (*N*=1,087). Their aim was to compare their EFA and CFA results with the model solution provided in the test manual and those models proposed for other versions of the WISC-V. Additionally, the authors compared bifactor model and higher-order model solutions as rival explanations and provided detailed information about all sources of decomposed factor variance and the according model-based reliability coefficients. In line with the aforementioned studies on standardization and clinical samples of international WISC-V versions, findings of Canivez et al. (2021) again supported a four-factor model rather than the five-factor model solution that was proposed by the test publishers. Furthermore, their CFA results additionally suggested a bifactor model with four group factors to be the best structural representation of the German WISC-V. The authors concluded that clinical interpretations are meaningful and therefore permissible regarding the FSIQ as a reliable estimate of the general factor. Due to the insufficient unique proportion of true score variance that is provided by each group factor as well as their weak model-based reliability, however, they also pointed out that drawing inferences from most of the primary index scores beyond the FSIQ (except for PSI) might result in misinterpretations.

Providing further insights into the structural validity of the German WISC-V are an indispensable requirement when it comes to evaluating the interpretability of test scores (AERA, APA, and NCME, 2014). In general, researchers as well as clinicians may only then rely on results of technically sound instruments with demonstrated validity for the population under evaluation (Evers et al., 2013). In order to make a substantial contribution to this field of research, the present study examined the latent factor structure of the 10 WISC-V primary subtests on an extended German standardization sample following both the EFA and the CFA approach. These approaches are also emphasized by the *ITC Guidelines for Translating and Adapting Tests* [International Test Commission (ITC) (2017)] as part of gold-standard test validation procedures. Both the EFA and CFA include the comparison between hierarchical and bifactor models as well as the examination of decomposed variance sources and model-based reliability. The present analyses can also be regarded as an extension of those conducted by Canivez et al. (2021) on the German WISC-V due to the following differences between both studies. First, Canivez et al. included all 15 WISC-V primary and secondary subtests in their analyses, whereas the present analyses are based on the 10 WISC-V primary subtests only. Given that primary subtests are more frequently administered in clinical practice, findings on the structural validity of the German WISC-V as well as the arising practical implications regarding the primary subtests might be of greater value for clinicians. Furthermore, the present EFA and CFA are conducted on the extended population-representative German standardization sample (*N*=1,646) and based on the original raw data, whereas all factor analyses reported by Canivez et al. were exclusively based on the correlation matrix of the original German standardization sample (*N*=1,087). Since the authors claimed that only little precision should be lost when using correlations that are rounded to two decimal places, results of the present analyses that are based on the raw data could then be used to support or refute this assertion. In due consideration of these differences, a replication of the results reported by Canivez et al. in the present study would at least speak to the robustness of the findings on the factor structure of the German WISC-V. Finally, results should then facilitate the evaluation of the German WISC-V test scores and the interpretive guidelines emphasized by the test publisher.

## Materials and Methods

### Participants and Sample Characteristics

For conducting EFA and CFA in the present study, data of a total of 1,646 children and adolescents (846 males; 800 females) aged between 6 and 16years were selected from an extended dataset of the nationally representative German WISC-V standardization sample. A detailed description of the standardization procedures is provided in the German WISC-V test manual (Wechsler, 2017b). The original standardization sample was obtained following stratified proportional sampling in order to match the recent German census for significant demographic variables including gender, age, parental education, type of school, and migration background. The total sample was bifurcated into separate EFA (*n*=823) and CFA (*n*=823) samples to match for gender, age, parental education, and migration background. Demographic characteristics of both samples are presented in Table 1.

**Table 1:** Demographic characteristics of the exploratory factor analyses (EFA) and confirmatory factor analyses (CFA) samples according to gender, age, parental education, and migration background.

	Gender	Age (years)				Parental education (%)					
		*N*	*M*	*SD*	Min/Max	0	1	2	3	4	Mig. (%)
**EFA sample**	Female	400	10.55	3.02	6/16	3.8	11.8	31.0	24.4	29.0	30.2
	Male	423	10.49	2.96	6/16	3.8	11.1	29.6	20.1	35.4	29.6
	Total	823	10.52	2.99	6/16	3.8	11.4	30.3	22.3	32.2	29.9
**CFA sample**	Female	400	10.53	3.02	6/16	3.6	11.3	31.3	25.3	28.7	30.5
	Male	423	10.52	2.98	6/16	3.1	10.9	28.6	21.5	35.9	29.3
	Total	823	10.52	3.00	6/16	3.3	11.1	29.9	23.4	32.3	29.9

Parental education is defined as the highest level of education achieved by either one parent or both (0 = no education, 1 = lower educational level, 2 = medium educational level, 3 = high educational level, and 4 = highest educational level). Mig. (%) = percentage of cases with migration background (migration background is indicated when either the child/adolescent or at least one parent is not born in Germany).

Matching quality was evaluated using Mann-Whitney and Kruskal-Wallis test statistics for non-parametric data. Besides equal gender distributions across the EFA and CFA samples, test statistics also indicated similar age distributions across both samples (*U*=338,241.00, *z*=−0.044, *p*=0.965). The EFA and CFA samples did not feature any significant differences in parental education (*χ*^2^ (1, *N*=1,646)=1.232, *p*=0.267), and the proportions of cases with migration background were the same for both samples. Univariate skewness and kurtosis estimation indicated no salient deviations from normal distributions for all subtest scores. When compared with the critical kurtosis value (kurt_c.r._), Mardia’s kurtosis estimates revealed multivariate non-normality for both the EFA sample (kurt=9.73>kurt_c.r._=9.01) and the CFA sample (kurt=7.27>kurt_c.r._=6.73). Test statistics also suggested that there were no significant mean differences in subtest scores between both samples, ranging from *t*=−0.20, *p*=0.84 (MR) to *t*=1.29, *p*=0.20 (CD). Since mean subtest scores and standard deviations appeared not to differ substantially from the expected population parameters, the EFA and CFA samples can be regarded as population-representative according to the stratification variables.

### Instrument and Measures

The German WISC-V was adapted from the original United States version (WISC-V US) based on a standardization kit that provides the basic framework for all European WISC-V versions. Like all Wechsler intelligence scales, the German WISC-V represents a comprehensive test of intelligence and provides a total of 15 subtests in its administration and scoring framework. Among those, there are 10 primary subtests including *Block Design* (BD), *Similarities* (SI), *Matrix Reasoning* (MR), *Digit Span* (DS), *Coding* (CD), *Vocabulary* (VC), *Figure Weights* (FW), *Visual Puzzles* (VP), *Picture Span* (PS), and *Symbol Search* (SS) from which seven primary subtests (BD, SI, MR, DS, CD, VC, and FW) are used to derive the FSIQ. Along with VP, PS, and SS, the scaled scores (*M*=10, *SD*=3) of all 10 primary subtests are required for calculating the five primary index scores for VCI, VSI, FRI, WMI, and PSI. Like the FSIQ, these index scores are defined by standard scores on the IQ scale (*M*=100, *SD*=15). All primary subtests of the German WISC-V feature good to excellent internal consistency coefficients, with Cronbach’s alpha ranging from 0.81 to 0.93.

### Data Analyses

Exploratory factor analyses (EFA) and confirmatory factor analyses (CFA) in the present study were based on the subtest covariance matrices of the bifurcated extended German WISC-V standardization sample. One covariance matrix was analyzed by conducting principal axis analyses on the EFA sample in order to assess the WISC-V factor structure using *SPSS 25* and the other was subjected to CFA for model comparisons using *AMOS 25*.

#### Exploratory Approach

To initially determine the number of factors to be extracted and retained in EFA, multiple criteria were examined to overcome the limitations of each single one. These criteria included Kaiser’s criterion of eigenvalues > 1, Cattell’s scree test, the standard error of scree (*SE*_scree_), Horn’s parallel analysis (HPA), Glorfeld’s modified parallel analysis (GPA), and the analysis of minimum average partials (MAP). While some criteria estimation algorithms were already implemented in *SPSS 25*, others had to be additionally computed with open source software. Scree analysis of standard errors was performed using the *SE*_scree_ program (Watkins, 2007) and random eigenvalues for HPA were produced using 100 iterations to provide stable eigenvalue estimates with the *Monte Carlo PCA for Parallel Analysis software* (Watkins, 2000). The *CIeigenvalue* program (Watkins, 2011) was used to calculate the 95% confidence intervals for eigenvalues that were then utilized for the GPA criterion. Since most extraction techniques tend to suggest the retention of only a few number of factors when there is a predominant general factor involved (Crawford et al., 2010), more complex factor solutions were also included in the analyses by forcing a predetermined number of factors to be extracted. These factor solutions were specified on a theoretical basis and evaluated in terms of their interpretability. Finally, principal axis analyses were used to examine the covariance matrix of the EFA sample and the retained factors were subjected to promax rotation for an oblique factor solution with *κ*=4 (Gorsuch, 1983) to allow for acceptably small correlations among factors. Following the recommendations made by Canivez and Watkins (2010), factors were required to have at least two salient subtest loadings (*λ*≥0.30) to be considered tenable.

Given that subtest scores reflect combinations of higher- and lower-order factors within the hierarchical WISC-V scoring framework, variance of the second-order factor had to be extracted first to residualize variance proportions from the first-order factors (Dombrowski et al., 2018). This way, relative variance apportionment could be examined separately for the first- and second-order factors. Estimating the unique variance proportions then allowed determining how good subtest indicators were represented by their respective factors. The Schmidt and Leiman procedure (SL procedure; Schmid and Leiman, 1957) has proven useful in accomplishing such a variance residualization and has already been applied in EFA studies on the WISC-V (Dombrowski et al., 2015, 2018; Canivez, 2016; Lecerf and Canivez, 2018). The SL procedure was applied to the hypothesized higher-order factor solutions (four-factor and five-factor EFA models) for comparison purposes and was conducted using the *MacOrtho program* (Watkins, 2004).

#### Confirmatory Approach

Different factor structures including both hierarchical and bifactor models were examined by conducting CFA on the 10 WISC-V primary subtests. The scale of latent variables was identified by fixing one loading of each factor to one (Keith, 2015). Subtest scaled scores (*M*=10, *SD*=3) were used after being initially checked for normality. As a rule of thumb, maximum likelihood (ML) estimation was considered adequate for data with an absolute value < 2 for skewness and an absolute value < 7 for kurtosis (West et al., 1995). Although the examination of univariate skewness and kurtosis did not reveal excessive deviation from normality for each single subtest score, however, Mardia’s multivariate kurtosis estimate indicated a salient leptokurtic distribution of subtest scores (kurt=7.27>kurt_c.r._=6.73). Since a significant departure from multivariate normality in ML estimation might lead to inflated *χ*^2^ test statistics and could thus result in an incorrect rejection of reasonable model solutions, the Bollen-Stine bootstrapping procedure was used to obtain adjusted *p* values for the likelihood ratio *χ*^2^ test statistics. As recommended by Nevitt and Hancock (2001), bootstrapping with 2,000 bootstrap samples should then sufficiently correct for any biasing impact that multivariate non-normality might have on the assessment of model fit (see Bollen and Long, 1993, for an overview). Finally, a total of five competing CFA models were specified according to those that have already been examined by the test publishers, starting with the least complex model that comprised the 10 WISC-V primary subtests loading only on one unidimensional *g*-factor (M_1_). All subsequent models with more than one first-order factor included *g* as the second-order factor. In addition to the latter, the second model (M_2_) comprised the two first-order factors *Verbal* (V) and *Performance* (P). In the third model (M_3_), the hierarchical structure was extended by *Verbal* (V), *Performance* (P), and *Processing Speed* (PS) as the first-order factors. The fourth model (M_4_) was composed of the four first-order factors *Verbal Comprehension* (VC), *Perceptual Reasoning* (PR), *Working Memory* (WM), and *Processing Speed* (PS), which have already been described for the WISC-IV (Wechsler, 2009). Finally, the WISC-V second-order five-factor model structure, including *Verbal Comprehension* (VC), *Visual-Spatial* (VS), *Fluid Reasoning* (FS), *Working Memory* (WM), and *Processing Speed* (PS), was specified by the fifth model (M_5_). CFA models with four- and five first-order factors were examined using both hierarchical (M_4_ and M_5_) and bifactor (M_6_ and M_7_) variants.

As with other versions of the WISC-V, some latent factors are underidentified in the five- and four-factor models because they provide only two subtest indicators. In line with other studies on this topic (e.g., Canivez et al., 2017; Watkins et al., 2017), identification of latent factor scales in CFA higher-order models was accomplished by setting one loading on each first-order factor and one loading on the second-order factor to one (see, Brown, 2015, for an overview). Additionally, all regression weights of error terms were set to one. In specifying latent factors with two subtest indicators in CFA bifactor models, the path coefficients on their group factors were constrained to equality prior to estimation to ensure identification (see, Little et al., 1999, for an overview).

A set of different model fit indexes was additionally analyzed for each single CFA model to improve evaluation. Due to the oversensitivity of *χ*^2^ for large sample sizes (Kline, 2016), the root mean square error of approximation (RMSEA) was jointly examined as an absolute fit index. RMSEA values of less than or equal to 0.01 indicate an excellent fit, a value of 0.05 corresponds to a good fit, and an RMSEA value of 0.10 should be used as an indicator for a poor fit (MacCallum et al., 1996). Following Chen’s (2007) recommendations, a considerable change in RMSEA values (ΔRMSEA>0.015) should also be taken into account when comparing the relative fit of competing CFA models. Additional relative fit indexes used were the comparative fit index (CFI) and the Tucker-Lewis index (TLI), where in both cases values above 0.95 correspond to a good fit (Hu and Bentler, 1999). Given that there are no universally accepted criteria for evaluating the overall model fit (McDonald, 2010), combinations of different heuristics were applied defining CFI and TLI≥0.90 along with RMSEA≤0.08 as a criterion for acceptable model fit and CFI and TLI≥0.95 in combination with RMSEA≤0.06 as a criterion for good model fit (Hu and Bentler, 1999). Finally, the Akaike information criterion (AIC) was used as an information-theoretic fit index to compare competing CFA models, with lower values indicating a better fit (Kaplan, 2000). Confirmatory factor analyses models were considered superior only if they indicated an adequate to good model fit and a substantially better fit (ΔCFI>0.01, ΔRMSEA>0.015, ∆AIC>10) than competing models (Chen, 2007).

Since Cronbach’s alpha is a limited reliability coefficient for multifactorial models (Yang and Green, 2011; Dunn et al., 2014), omega-hierarchical (ω_H_) and omega-hierarchical subscale (ω_HS_) were additionally estimated to provide meaningful model-based reliability coefficients (Reise, 2012; Rodriguez et al., 2016; Watkins, 2017). The ω_H_ coefficient represents an estimate for the general factor reliability independent of the group factor variances, whereas ω_HS_ indicates the reliability of each group factor that is adjusted for all other group and general factor variances (Brunner et al., 2012). Therefore, ω_HS_ may control for that proportion of reliability attributable to the general factor and is useful for judging the informative value of each single first-order factor score. Robust ω_HS_ coefficient could thus indicate that most of the reliable index variance are rather independent of the FSIQ, so that individual cognitive functioning may be meaningfully interpreted on the index level. On the contrary, low values of ω_HS_ would suggest that most of the reliable variance of the indexes are attributable to the FSIQ. This might then compromise the interpretability of index scores as unambiguous indicators of specific cognitive domains (Rodriguez et al., 2016). For ω_H_ and ω_HS_, it has been recommended that values near 0.75 should be preferred and values should not be less than 0.50 (Reise et al., 2013). Omega coefficients were supplemented by the construct replicability coefficient *H* to evaluate how adequate the latent variables were represented by their related indicators (Hancock and Mueller, 2001). *H* values should not be less than 0.70 in order to ensure a high quality of indicators and replicability of latent variables. The *Omega program* (Watkins, 2013) was used to obtain all omega coefficients, *H* coefficients, and other sources of variance (see Zinbarg et al., 2006; Brunner et al., 2012, for an overview).

## Results

### Exploratory Approach

The KMO test statistic indicated an adequate sampling as its value of 0.88 exceeded the minimum criterion of 0.60 (Kaiser, 1974). Since Bartlett’s test of sphericity indicated that the correlation matrix diverged significantly from the identity matrix (*χ*^2^ (45)=3,079.938, *p*<0.001) and initial communality estimates ranged from 0.300 to 0.550 prior to extraction, the covariance matrix was considered appropriate for conducting EFA.

#### Factor Extraction

Cattell’s scree test suggested two factors to be extracted as the scree plot revealed two factors that met Kaiser’s criterion of eigenvalues > 1, for which the *SE*_scree_ also indicated nontrivial standard errors of estimation. Two factors with eigenvalues significantly greater than randomly generated eigenvalues were also identified in GPA, HPA, and MAP, thus supporting the retention of two factors in total. Since it has been recommended for principal axis analysis to better over-extract than under-extract (Wood et al., 1996), traditionally promoted Wechsler model structures were also considered by additionally forcing the extraction of three, four, and five factors in EFA. Five factors were extracted at first to examine the WISC-V factor structure that has been proposed by the test publishers. Alternative model solutions were then sequentially examined for adequacy in descending order by the number of factors from four to two factors.

#### EFA Model Solutions

##### Five-Factor Model Solution (Oblique)

As presented in Table 2, the extraction of five factors produced one factor with only one salient factor pattern coefficient. According to Kaufman’s criteria (1994), a weak *g*-loading indicated only a poor association between CD and a general factor (*S_g_*=0.488). While each of four subtest pairs, including SI and VC (*Verbal Comprehension*), BD and VP (*Visual Spatial*), DS and PS (*Working Memory*), and CD and SS (*Processing Speed*), saliently loaded on a corresponding common factor, MR and FW appeared to share insufficient common variance to constitute a single factor for *Fluid Reasoning*. FW completely failed to provide a salient loading on any factor and the very high factor pattern coefficient for MR (*P*=0.921) further supported the redundancy of a separate factor for *Fluid Reasoning*. Given that no reasonable factor could be found for *Fluid Reasoning*, the five-factor model was judged inadequate due to over-extraction.

**Table 2:** Exploratory factor analysis of the 10 WISC-V primary substests: Five-factor model solution (oblique) with promax rotation for the EFA sample (*n*=823).

WISC-V subtest	*S_g_*	F1		F2		F3		F4		F5		*h* ^2^
		*P*	*S*	*P*	*S*	*P*	*S*	*P*	*S*	*P*	*S*	
SI	0.730	**0.642**	0.778	0.011	0.566	0.068	0.586	0.107	0.619	0.026	0.390	0.622
VC	0.712	**0.907**	0.892	−0.021	0.524	−0.079	0.506	−0.063	0.546	−0.016	0.346	0.808
BD	0.679	0.061	0.530	**0.409**	0.671	0.166	0.609	0.093	0.582	0.071	0.409	0.492
VP	0.751	−0.025	0.554	**0.967**	0.896	−0.059	0.626	−0.002	0.602	−0.023	0.400	0.806
MR	0.697	−0.048	0.509	−0.025	0.596	**0.921**	0.844	−0.029	0.612	−0.013	0.337	0.717
FW	0.645	0.178	0.555	0.147	0.566	0.230	0.601	0.209	0.595	−0.024	0.323	0.436
DS	0.649	0.058	0.522	−0.028	0.512	0.140	0.588	**0.560**	0.694	0.016	0.359	0.492
PS	0.566	−0.039	0.430	0.029	0.456	−0.096	0.466	**0.752**	0.669	−0.008	0.312	0.453
CD	0.488	−0.044	0.315	−0.063	0.336	−0.068	0.313	0.181	0.413	**0.738**	0.748	0.570
SS	0.501	0.035	0.341	0.051	0.382	0.048	0.336	−0.163	0.327	**0.815**	0.796	0.643
Eigenvalue		4.58		0.79		0.58		0.73		1.20		
% Variance		45.82		7.89		5.78		7.28		11.95		
Factor correlations												
		F1		F2		F3		F4		F5		
F1		1		–		–		–		–		
F2		0.650		1		–		–		–		
F3		0.650		0.736		1		–		–		
F4		0.686		0.700		0.757		1		–		
F5		0.433		0.475		0.431		0.484		1		

SI = Similarities; VC = Vocabulary; BD = Block Design; VP = Visual Puzzles; MR = Matrix Reasoning; FW = Figure Weights; DS = Digit Span; PS = Picture Span; CD = Coding; and SS = Symbol Search. F1, Verbal Comprehension; F2, Visual Spatial; F3, Fluid Reasoning; F4, Working Memory; and F5, Processing Speed. *P*, Pattern Coefficient (factor loading); *S*, Structure Coefficient (factor correlation); and *h*^2^, Communality (after extraction). General structure coefficients (S*_g_*) are based on the factor coefficients for the first unrotated factor (*g*-factor loadings) and eigenvalues. Salient factor loadings (≥ 0.30) are indicated in bold numbers.

##### Four-Factor Model Solution (Oblique)

When extracting four WISC-V factors, *g*-loadings ranged from *S_g_*=0.495 (SS) to *S_g_*=0.736 (SI) and were, except for SS, within the fair to good range based on Kaufman’s criteria. As Table 3 illustrates, the four-factor model solution provided stable and well-defined factors with theoretically consistent factor patterns. Thus, robust factor pattern coefficients were found for Verbal Comprehension, including SI (*P*=0.624) and VC (*P*=0.948) and for *Perceptual Reasoning*, including BD (*P*=0.692), VP (*P*=0.805), MR (*P*=0.461), and FW (*P*=0.328). This was also the case for *Working Memory*, including reasonable pattern coefficients for DS (*P*=0.686) and PS (*P*=0.581), as well as for *Processing Speed* with strong pattern coefficients for CD (*P*=0.813) and SS (*P*=0.710). Since none of the subtests featured salient loadings on more than one factor, simple structure could be established for this model solution.

**Table 3:** Exploratory factor analysis of the 10 WISC-V primary subtests: four-factor model solution (oblique) with promax rotation for the EFA sample (*n*=823).

WISC-V subtest	*S_g_*	F1		F2		F3		F4		*h* ^2^
		*P*	*S*	*P*	*S*	*P*	*S*	*P*	*S*	
SI	0.736	**0.624**	0.780	0.080	0.624	0.137	0.627	0.023	0.368	0.632
VC	0.713	**0.948**	0.884	−0.038	0.569	−0.054	0.550	−0.006	0.324	0.785
BD	0.690	−0.003	0.510	**0.692**	0.735	0.028	0.576	0.050	0.386	0.543
VP	0.721	−0.004	0.535	**0.805**	0.788	−0.026	0.590	0.011	0.375	0.621
MR	0.662	−0.025	0.490	**0.461**	0.669	0.222	0.637	−0.042	0.310	0.488
FW	0.650	0.138	0.541	**0.328**	0.624	0.289	0.613	−0.040	0.299	0.445
DS	0.658	0.018	0.503	0.031	0.570	**0.686**	0.726	0.010	0.342	0.528
PS	0.560	−0.011	0.417	0.044	0.489	**0.581**	0.617	0.021	0.301	0.382
CD	0.504	−0.034	0.304	−0.120	0.352	0.151	0.404	**0.813**	0.811	0.668
SS	0.495	0.042	0.330	0.160	0.407	−0.150	0.321	**0.710**	0.734	0.552
Eigenvalue		1.20		4.58		0.79		0.73		
% Variance		11.95		45.82		7.89		7.28		
Factor correlations										
		F1		F2		F3		F4		
F1		1		–		–		–		
F2		0.686		1		–		–		
F3		0.670		0.762		1		–		
F4		0.393		0.469		0.453		1		

SI = Similarities; VC = Vocabulary; BD = Block Design; VP = Visual Puzzles; MR = Matrix Reasoning; FW = Figure Weights; DS = Digit Span; PS = Picture Span; CD = Coding; and SS = Symbol Search. F1, Verbal Comprehension; F2, Perceptual Reasoning; F3, Working Memory; and F4, Processing Speed. P, Pattern Coefficient (factor loading); S, Structure Coefficient (factor correlation); and *h*^2^, Communality (after extraction). General structure coefficients (S*_g_*) are based on the factor coefficients for the first unrotated factor (*g*-factor loadings). Salient factor loadings (≥ 0.30) are indicated in bold numbers.

##### Three- and Two-Factor Model Solutions (Oblique)

EFA results for the model solutions with three and two extracted factors are presented in Table 4. The factors for *Perceptual Reasoning* and *Working Memory* from the four-factor model solution merged into one factor labeled as *Performance* (P), while *Verbal Comprehension* and *Processing Speed* remained intact in the three-factor model. For the sake of theoretical consistency, Verbal Comprehension was relabeled as Verbal and there were no salient cross-loadings on multiple factors among the subtests in the three-factor model solution. The latter also applied to the two-factor model solution, in which only *Processing Speed* remained distinct and intact. Eight out of ten subtests appeared to load on another single factor, which was labeled as *General Intelligence (g)* due to its complexity, and the patterns of subtest associations in both models clearly indicated a conflation of theoretically meaningful constructs. Since this is an already well-known phenomenon of under-extraction in EFA, the three- and two-factor model solutions were both considered inadequate.

**Table 4:** Exploratory factor analysis of the 10 WISC-V primary subtests: three- and two-factor model solutions (oblique) with promax rotation for the EFA sample (*n*=823).

WISC-V subtest	Three-factor model solution (oblique)								Two-factor model solution (oblique)					
	*S_g_*	F1 (V)		F2 (P)		F3 (PS)		*h* ^2^	*S_g_*	F1 (*g*)		F2 (PS)		*h* ^2^
		*P*	*S*	*P*	*S*	*P*	*S*			*P*	*S*	*P*	*S*	
SI	0.739	**0.598**	0.774	0.237	0.671	0.021	0.384	0.629	0.726	**0.740**	0.738	−0.005	0.379	0.544
VC	0.717	**0.926**	0.888	−0.049	0.602	−0.008	0.340	0.790	0.664	**0.687**	0.676	−0.020	0.337	0.458
BD	0.685	−0.015	0.493	**0.686**	0.705	0.055	0.404	0.499	0.687	**0.650**	0.687	0.070	0.408	0.475
VP	0.709	−0.005	0.517	**0.725**	0.733	0.022	0.395	0.538	0.710	**0.696**	0.715	0.037	0.399	0.513
MR	0.667	−0.055	0.472	**0.773**	0.708	−0.050	0.327	0.505	0.666	**0.694**	0.680	−0.028	0.333	0.462
FW	0.653	0.117	0.528	**0.607**	0.667	−0.046	0.315	0.452	0.658	**0.699**	0.675	−0.046	0.317	0.457
DS	0.646	0.052	0.493	**0.612**	0.659	0.019	0.357	0.436	0.650	**0.645**	0.657	0.022	0.357	0.432
PS	0.554	0.016	0.411	**0.543**	0.568	0.026	0.313	0.323	0.557	**0.546**	0.561	0.029	0.313	0.316
CD	0.500	−0.023	0.300	0.004	0.398	**0.793**	0.786	0.618	0.502	−0.002	0.396	**0.767**	0.766	0.586
SS	0.497	0.025	0.321	−0.001	0.396	**0.735**	0.745	0.555	0.505	0.007	0.401	**0.760**	0.763	0.582
Eigenvalue		1.20		4.58		0.79			4.58			1.20		
% Variance		11.95		45.82		7.89			45.82			11.95		
Factor correlations														
		F1 (V)		F2 (P)		F3 (PS)			F1 (*g*)			F2 (PS)		
F1		1		–		–		F1	–			–		
F2		0.708		1		–		F2	0.519			–		
F3		0.403		0.517		1								

SI = Similarities; VC = Vocabulary; BD = Block Design; VP = Visual Puzzles; MR = Matrix Reasoning; FW = Figure Weights; DS = Digit Span; PS = Picture Span; CD = Coding; and SS = Symbol Search. F1 (V), Verbal; F2 (P), Performance; F3 (PS), Processing Speed; F1 (*g*), General Intelligence; F2 (PS), and Processing Speed. P, Pattern Coefficient (factor loading); S, Sructure Coefficient (factor correlation); and *h*^2^, Communality (after extraction). General structure coefficients (S*_g_*) are based on the factor coefficients for the first unrotated factor (*g*-factor loadings). Salient factor loadings (≥ 0.30) are indicated in bold numbers.

##### Higher-Order Four-Factor Model Solutions (SL Orthogonalized)

According to the present results, the four-factor model solution appeared to be the most reasonable and appropriate among all EFA models examined. Therefore, it was subjected to higher-order EFA and orthogonalized using the SL procedure. As displayed in Table 5, all 10 subtests were exclusively associated with their theoretically suggested factors as proposed for the WISC-IV. When analyzing the sources of variance, *g* appeared to account for 29.9% of the total variance and 56.5% of the common variance. It also explained between 19.4% (PS) and 39.6% (SI) of the individual subtest variance. A total of 53% of the subtest score variances could be explained by *g* and group factors combined, leaving 47% unique or error variance unexplained. Among all subtests, FW accounted for the smallest proportion of common variance (29.4%), thus being mostly influenced by unique variance (70.6%).

**Table 5:** Sources of variance in the 10 WISC-V primary subtests for the EFA sample (*n*=823) according to a SL-orthogonalized higher-order factor model with four first-order factors.

WISC-V subtest	*g*		F1		F2		F3		F4		*h* ^2^	*u* ^2^
	*b*	*S* ^2^	*b*	*S* ^2^	*b*	*S* ^2^	*b*	*S* ^2^	*b*	*S* ^2^		
SI	0.629	0.396	**0.422**	**0.178**	0.055	0.003	0.099	0.010	0.016	0.000	0.574	0.426
VC	0.628	0.394	**0.642**	**0.412**	−0.026	0.001	−0.039	0.002	−0.004	0.000	0.807	0.193
BD	0.559	0.312	−0.002	0.000	**0.472**	**0.223**	0.020	0.000	0.035	0.001	0.535	0.465
VP	0.576	0.332	−0.003	0.000	**0.549**	**0.301**	−0.019	0.000	0.008	0.000	0.633	0.367
MR	0.512	0.262	−0.017	0.000	**0.324**	**0.105**	0.161	0.026	−0.029	0.001	0.367	0.633
FW	0.441	0.194	0.093	0.009	**0.315**	**0.099**	0.209	0.044	−0.028	0.001	0.294	0.706
DS	0.516	0.266	0.012	0.000	0.021	0.000	**0.497**	**0.247**	0.007	0.000	0.513	0.487
PS	0.440	0.194	−0.007	0.000	0.030	0.001	**0.421**	**0.177**	0.015	0.000	0.371	0.629
CD	0.578	0.334	−0.023	0.001	−0.082	0.007	0.109	0.011	**0.563**	**0.317**	0.651	0.349
SS	0.556	0.309	0.028	0.001	0.109	0.012	−0.109	0.011	**0.492**	**0.242**	0.551	0.449
Total *S*^2^		0.299		0.059		0.073		0.042		0.056	0.530	0.470
ECV		0.565		0.111		0.138		0.080		0.106		
ω		0.883		0.814		0.766		0.612		0.750		
ω_H_/ω_HS_		0.737		0.340		0.297		0.293		0.348		
Relative ω		0.835		0.417		0.387		0.480		0.464		
*H*		0.815		0.479		0.486		0.352		0.439		
PUC		0.800										

SI = Similarities; VC = Vocabulary; BD = Block Design; VP = Visual Puzzles; MR = Matrix Reasoning; FW = Figure Weights; DS = Digit Span; PS = Picture Span; CD = Coding; and SS = Symbol Search. *g*, General Intelligence (second-order factor); F1, Verbal Comprehension (first-order factor); F2, Perceptual Reasoning (first-order factor); F3, Working Memory (first-order factor); and F4, Processing Speed (first-order factor). *b*, Factor Loading; S^2^, Explained Variance; *h*^2^, Communality; *u*^2^, Unique Variance; ECV, Explained Common Variance; ω, Omega Coefficient; ω_H_, Omega-Hierarchical Coefficient (general factor); ω_HS_, Omega-Hierarchical Coefficient (group factors); *H*, Replicability Index (construct reliability); and PUC, Percentage of Uncontaminated Correlations. Salient factor loadings (≥ 0.30) and variance estimates are indicated in bold numbers.

While the ω_H_ coefficient value of 0.737 indicated that the second-order factor (*g*) was precisely measured and barely influenced by variances in other factors, all ω_HS_ coefficient values for the first-order factors, ranging from ω_HS_=0.293 (WM) to ω_HS_=0.348 (PS), fell below the required minimum criterion of ω_HS_=0.500 (Reise et al., 2013). Thus, model-based reliability coefficients suggest that an overall measure, such as the FSIQ, could appear reliable for scale interpretation, whereas unit-weighted composite scores based on the four indexes might contain too little true score variance for meaningful interpretation. This was also supported by the construct replicability coefficients. While the *H* coefficient value for the second-order factor (*H*=0.815) indicated that *g* was well defined by the subtest indicators, all *H* coefficient values for the first-order factors, ranging from *H*=0.352 (WM) to *H*=0.486 (PR), failed to meet the required minimum criterion of *H*=0.700 (Hancock and Mueller, 2001; Rodriguez et al., 2016). Given that the first-order factors appeared to be insufficiently defined by their associated subtest indicators, the WISC-V primary subtests cannot be suggested to produce consistent scores on the four indexes across measurements.

### Confirmatory Approach

Results of the confirmatory factor analyses (CFA) based on maximum likelihood estimation and the according model fit statistics are presented in Table 6. As indicated by the fit indexes, the unidimensional *g*-factor model (M_1_) and the second-order two-factor model (M_2_) were found to inadequately represent the empirical data. This was due to unacceptably low CFI and TLI values (< 0.90) as well as too high RMSEA values (> 0.08) for the minimum fit criteria. However, CFI and TLI values (> 0.90) as well as RMSEA values (< 0.08) indicated at least acceptable fit for the second-order three-factor model (M_3_). The second-order four-factor model (M_4_) and the second-order five-factor model (M_5_) both appeared to represent well-fitting CFA models to the data (CFI>0.95; TLI>0.95; RMSEA<0.06). When comparing the fit of both models with M_3_, M_4_ (ΔCFI=0.033, ΔRMSEA=0.036, ΔAIC=101.765) and M_5_ (ΔCFI=0.039, ΔRMSEA=0.047, ΔAIC=118.458) were found to be superior. Only with respect to the difference in AIC, M_5_ fitted the data slightly better than M_4_ (ΔAIC=16.693). Likewise, the bifactor model with four group factors (M_6_) and the bifactor model with five group factors (M_7_) were found to be well-fitting models as well. When comparing both higher-order models with the corresponding bifactor models, the bifactor model with four group factors (M_6_) turned out to slightly surpass the second-order four-factor model (M_4_) but only according to the lower AIC (ΔAIC=16.905). Due to its local underidentification, the second-order five-factor model (M_5_) was mathematically equivalent to the corresponding bifactor model (M_7_), thus featuring the same fit statistics.

**Table 6:** Maximum likelihood estimation and model fit statistics based on the 10 WISC-V primary subtests for the CFA sample (*n*=823).

CFA model	Indexes of model fit						
	*χ* ^2^	*df*	CFI	TLI	RMSEA	(90% CI)	AIC
M_1_: g	472.540	35	0.857	0.816	0.123	[0.114, 0.133]	512.540
M_2_: V, P	398.097	34	0.881	0.842	0.114	[0.104, 0.124]	440.097
M_3_: V, P, PS	167.417	32	0.956	0.938	0.072	[0.061, 0.083]	213.417
M_4_: VC, PR, WM, PS	63.652	31	0.989	0.984	0.036	[0.023, 0.048]	111.652
M_5_: VC, VS, FR, WM, PS	44.959	30	0.995	0.993	0.025	[0.006, 0.039]	94.959
**M_6_: VC, *VS*, FR, WM, PS^1^**	**40.747**	**28**	**0.996**	**0.993**	**0.024**	**[0.001, 0.038]**	**94.747**
M_7_: VC, *VS*, VC, VS, FR, WM, PS^2^ ^3^	44.959	30	0.995	0.993	0.025	[0.006, 0.039]	94.959

1 Subtest loadings on the underindentified factors VC, WM, and PS were constrained to be equal due to model identification.

2 Subtest loadings on the underindentified factors VC, VS, FR, WM, and PS were constrained to be equal due to model identification.

3 Model is mathematically equivalent to higher-order model M_5_ due to constraining each factor’s loading to equality.

General intelligence (g) is the second-order factor in all higher-order models, which are specified according to those reported in the WISC-V Technical and Interpretive Manual. M_1_ = unidimensional g-factor model, M_2_ = second-order two-factor model with Verbal (V) and Performance (P) as first-order factors, M_3_ = second-order three-factor model with Verbal (V), Performance (P), and Processing Speed (PS) as first-order factors, M_4_ = second-order four-factor model with Verbal Comprehension (VC), Perceptual Reasoning (PR), Working Memory (WM), and Processing Speed (PS) as first-order factors, M_5_ = second-order five-factor model with Verbal Comprehension (VC), Visual Spatial (VS), Fluid Reasoning (FR), Working Memory (WM), and Processing Speed (PS) as first-order factors, M_6_ = bifactor model with g as the general factor and VC, PR, WM, and PS as group factors, M_7_ = bifactor model with g as the general factor and VC, VS, FR, WM, and PS as group factors. CFI = comparative fit index, TLI = Tucker-Lewis index, RMSEA = root mean square error of approximation, (90% CI) = confidence interval for RMSEA, AIC = Akaike’s information criterion. 1 Subtest loadings on the underindentified factors VC, WM, and PS were constrained to be equal due to model identification. 2 Subtest loadings on the underindentified factors VC, VS, FR, WM, and PS were constrained to be equal due to model identification. 3 Model is mathematically equivalent to higher-order model M_5_ due to constraining each factor’s loading to equality.

Even though the bifactor model with four group factors (M_6_) provided the most favorable fit statistics, there was no meaningful difference in CFI, TLI, RMSEA, and AIC values between M_6_ and the two models including five factors (M_5_ and M_7_). However, it should be noted that M_6_ was the only CFA model that featured an acceptable fit to the data according to its likelihood ratio *χ*^2^ statistic with adjusted *p* values (*χ*^2^=40.747, *df*=28, *p*=0.06). Finally, the bifactor model with four group factors (M_6_) was considered the best fitting model in the present CFA when taking all goodness-of-fit indexes into account.

**Figure 1:**
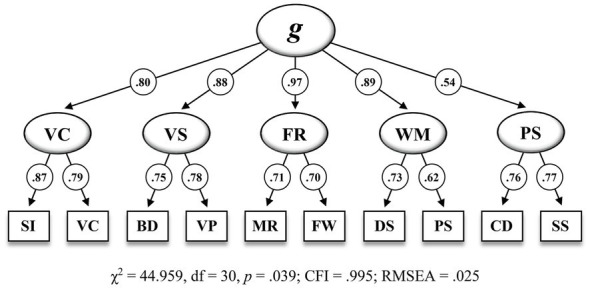
Second-order five-factor model including standardized estimations for the CFA sample (n = 823) on the 10 WISC-V primary subtests (M_6_ in Table 6). SI = Similarities, VC = Vocabulary, BD = Block Design, VP = Visual Puzzles, MR = Matrix Reasoning, FW = Figure Weights, DS = Digit Span, PS = Picture Span, CD = Coding, SS = Symbol Search. *g*, = General Intelligence, VC = Verbal Comprehension, VS = Visual Spatial, FR = Fluid Reasoning, WM = Working Memory, PS = Processing Speed. All standardized parameter estimates are significant at *p*<0.001.

**Figure 2:**
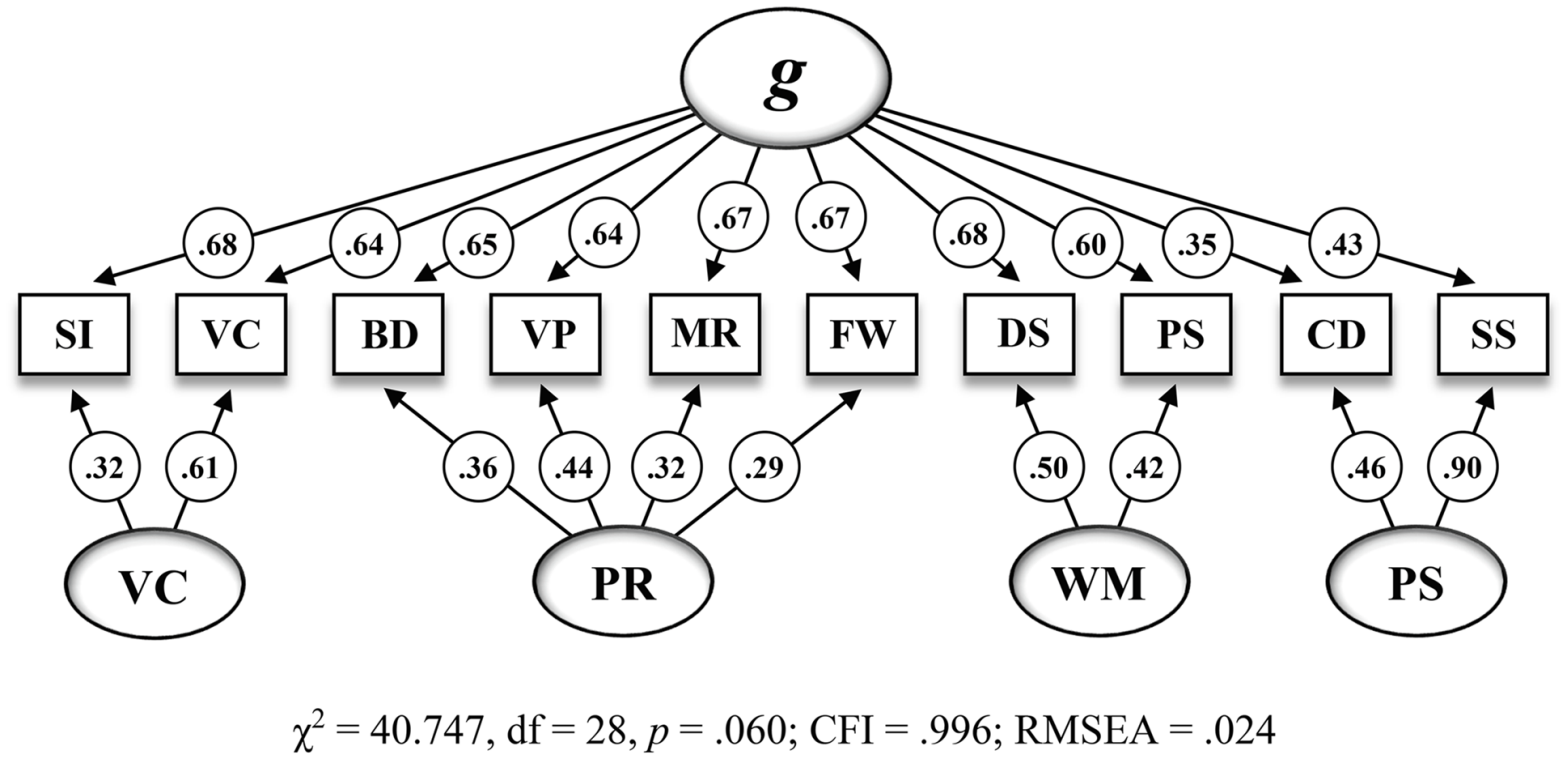
Bifactor model with four group factors including standardized estimations for the CFA sample (*n*=823) on the 10 WISC-V primary subtests (M_6_ in Table 6). SI = Similarities, VC = Vocabulary, BD = Block Design, VP = Visual Puzzles, MR = Matrix Reasoning, FW = Figure Weights, DS = Digit Span, PS = Picture Span, CD = Coding, SS = Symbol Search. *g* = General Intelligence, VC = Verbal Comprehension, PR = Perceptual Reasoning, WM = Working Memory. All standardized parameter estimates are significant at *p*<0.05.

Figure 1 shows the second-order five-factor model (M_5_) that has been proposed by the test publishers and Figure 2 shows the bifactor model with four group factors (M_6_) that appeared to be the best fitting model in the present CFA.

As shown in Table 7, all subtest indicators featured significant and reasonable loadings on *g* and on their related group factors in M_6_. However, decomposed variance estimates further indicated that smaller proportions of explained common variance (ECV), ranging from ECV=0.069 (WM) to ECV=0.164 (PS), were uniquely associated with the group factors than those associated with *g*. Conclusively, about 60.7% of the explained common variance in the subtest indicators appeared to be uniquely attributable to *g*. Consistent with the ECV estimates, the analysis of model-based reliability revealed rather small ω_HS_ coefficient values for the group factors, ranging from ω_HS_=0.187 (PR) to ω_HS_=0.589 (PS), when compared to the ω_H_ coefficient value of 0.794 for *g*. While *g* turned out to be precisely measured by the subtest indicators, most of the ω_HS_ coefficient values for the group factors were below the required minimum criterion of ω_HS_=0.500 (Reise et al., 2013). As in the case of the higher-order four-factor EFA model, unit-weighted composite scores for three out of four indexes might therefore produce too little true score variance to fully recommend interpretation.

**Table 7:** Sources of variance in the 10 WISC-V primary subtests for the CFA sample (*n*=823) according to a bifactor model with four group factors (M_6_).

WISC-V subtest	*g*		F1		F2		F3		F4		*h* ^2^	*u* ^2^
	*b*	*S* ^2^	*b*	*S* ^2^	*b*	*S* ^2^	*b*	*S* ^2^	*b*	*S* ^2^		
SI	0.683	0.466	0.321	0.103							0.570	0.430
VC	0.637	0.406	0.611	0.373							0.779	0.221
BD	0.652	0.425			0.359	0.129					0.554	0.446
VP	0.637	0.406			0.444	0.197					0.603	0.397
MR	0.667	0.445			0.322	0.104					0.549	0.451
FW	0.674	0.454			0.288	0.083					0.537	0.463
DS	0.684	0.468					0.497	0.247			0.715	0.285
PS	0.604	0.365					0.421	0.177			0.542	0.458
CD	0.347	0.120							0.462	0.213	0.334	0.666
SS	0.433	0.187							0.895	0.801	0.989	0.011
Total *S*^2^		0.374		0.048		0.051		0.042		0.101	0.617	0.383
ECV		0.607		0.077		0.083		0.069		0.164		
ω		0.916		0.800		0.835		0.771		0.783		
ω_H_/ω_HS_		0.794		0.266		0.187		0.260		0.589		
Relative ω		0.867		0.333		0.224		0.337		0.752		
*H*		0.865		0.415		0.375		0.352		0.811		
PUC		0.800										

SI = Similarities; VC = Vocabulary; BD = Block Design; VP = Visual Puzzles; MR = Matrix Reasoning; FW = Figure Weights; DS = Digit Span; PS = Picture Span; CD = Coding; and SS = Symbol Search. *g*, General Intelligence (general factor); F1, Verbal Comprehension (group factor); F2, Perceptual Reasoning (group factor); F3, Working Memory (group factor); and F4, Processing Speed (group factor). *b*, Factor Loading (significant at *p*<0.05), *S*^2^, Explained Variance; *h*^2^, Communality; *u*^2^, Unique Variance; ECV, Explained Common Variance; ω, Omega Coefficient; ω_H_, Omega-Hierarchical Coefficient (general factor); ω_HS_, Omega-Hierarchical Coefficient (group factors); *H*, Replicability Index (construct reliability); and PUC, percentage of uncontaminated correlations.

While the construct replicability coefficient *H* in Table 7 suggested that *g* was well defined by the 10 subtest indicators, *H* coefficient values for the other factors, ranging from 0.352 (WM) to 0.811 (PS), indicated that three out of four group factors were not adequately defined by their subtest indicators. This means that, with the exception of PSI, eight out of 10 WISC-V primary subtests would not produce consistent scores on their related indexes VCI, PRI, and WMI across measurements.

## Discussion

The overall factor structure of the WISC-V has been a subject of controversial debate to this very day. Even though the test publishers claimed support for the second-order five-factor model based on CFA analyses, this model could not be fully replicated in a variety of different standardization samples (e.g., Canivez et al., 2017; Watkins et al., 2017; Fenollar-Cortés and Watkins, 2018; Lecerf and Canivez, 2018). Therefore, the major aim of the present study was to determine structural validity of the German WISC-V. Since CFA results provided in the test manual have been frequently criticized by researchers, different factor analytical procedures were conducted on the large extended and bifurcated German standardization sample.

In line with the results reported by Canivez et al. (2021), EFA failed to replicate five valid factors for the 10 primary subtests of the German WISC-V, thus suggesting psychometric inappropriateness of the second-order five-factor model. When forced to extract more than one factor, EFA results suggested a four-factor model rather than a five-factor model, particularly because the *Fluid Reasoning* (FR) factor failed to provide more than one salient subtest indicator. *Visual spatial* (VS) and *Fluid Reasoning* (FR) were not found to emerge as distinct factors, as their related subtest indicators shared variance with only one single factor similar to *Perseptual Reasoning* (PR) in the WISC-IV framework. Moreover, hierarchical EFA with the SL orthogonalization indicated superiority of the second-order factor over the first-order factors, as *g* accounted for between 4 and 7 times as much common subtest variance as any single first-order factor and more common subtest variance than all four first-order factors combined. Model-based reliability estimates for the first-order factors appeared to be low in value when compared to the reliability coefficient for the second-order factor. Since construct replicability estimates were also found to be unacceptably low for each of the first-order factors, the according WISC-V indexes may be considered limited in measuring unique cognitive dimensions (Brunner et al., 2012; Reise, 2012; Rodriguez et al., 2016). Since Canivez et al. reported similar findings when analyzing the entire set of 15 primary and secondary subtests of the German WISC-V, the present EFA and CFA results can be considered as meaningful and robust.

Consistent with the EFA results, a four-factor structure was judged as being the best fitting model in CFA. While the second-order and bifactor five-factor model solutions both provided acceptable fit to the data, fit indexes tended to slightly favor four factors within a bifactor structure. This was also supported by the subtest indicators, each of which was for the most part saliently loading on just one associated group factor and thus achieving the preferable simple structure. As with the SL-orthogonalized EFA model, the assessment of variance sources again indicated the dominance of *g* on the one hand and the limited unique measurement of the first-order factors on the other hand. While about 7% (WM) to 16% (PS) of the common subtest variance could be explained by the according first-order factors, *g* accounted for nearly 61% of the common subtest variance. In particular, *g* accounted for between three and eight times as much common subtest variance as any single first-order factor and about 1.5 times as much common variance as all four first-order factors combined. Consistent with the findings of Canivez et al. (2021), the present EFA and CFA results were not surprising as it has previously been reported that the greatest proportions of common variance are associated with the second-order factor and smaller proportions of common variance are apportioned to the first-order factors (Canivez et al., 2016, 2017; Watkins et al., 2017; Fenollar-Cortés and Watkins, 2018; Lecerf and Canivez, 2018). When analyzing model-based reliability and construct replicability according to the bifactor four-factor model, CFA results were consistent with what was already indicated by EFA. In both analyses, model-based reliability and construct replicability coefficients for *g* turned out to be satisfactory, thus suggesting a confident individual interpretation of an overall measure, such as the FSIQ. Except for PS, however, reliability and replicability coefficients for the group or first-order factors appeared to be too low to suggest that the according unique cognitive dimensions are sufficiently well represented by the WISC-V primary indexes. Similar results have not only been found for the German WISC-V as described earlier (Canivez et al., 2021) but had already been observed in studies focusing on international versions of the WISC-IV (Watkins, 2010; Canivez, 2014; Canivez et al., 2017) and on other Wechsler scales as well (Canivez and Watkins, 2010; Golay and Lecerf, 2011; Watkins and Beaujean, 2014). Even though results of the present study indicated meaningful interpretation of an overall measure for *g*, this is only true if the corresponding composite score is based on all 10 primary subtests. Given that the FSIQ is calculated using only seven out of 10 primary subtest scores in all versions of the WISC-V, this measure could likely under- or over-estimate true levels of general intellectual ability. Therefore, psychometric appropriateness and interpretability of *g* cannot be equally guaranteed for or at least not fully transferred to the FSIQ.

Furthermore, the present findings do not only provide substantial support for the results recently reported by Canivez et al. (2021), they also underline the need of comparing hierarchical and non-hierarchical model structures when analyzing the structural validity of the WISC-V. In hierarchical models of intelligence, the second-order factor is only indirectly connected to the subtest indicators as the first-order factors are suggested to fully mediate these associations. In bifactor models, by contrast, the general factor and the group factors both directly influence the subtest indicators at the same level of inference (Gignac, 2008). Consequently, direct subtest associations are easier to interpret as general and specific subtest influences can be simultaneously examined. However, there are still varying perspectives on which of the two model solutions are the most appropriate representation of intelligence (e.g., Brunner et al., 2012; Gignac and Watkins, 2013; Beaujean, 2015; Frisby and Beaujean, 2015; Reynolds and Keith, 2017). While some researchers suggested that bifactor models in general benefit from statistical biases related to unmodeled complexity (Murray and Johnson, 2013) and unique constraints within hierarchical model solutions (Mansolf and Reise, 2017), others have not found such biases exclusively favoring bifactor models Canivez et al., 2017. Even though Canivez et al. (2018) claimed that *post-hoc* model modifications with a lack of conceptual grounding (e.g., cross-loadings and correlated error terms) are often added by researchers preferring hierarchical model solutions to only improve model fit, such *post-hoc* model modifications are not restricted to analyses of hierarchical models only. Bifactor models may as well over-estimate loadings on a higher-order factor while under-estimating variance accounted for lower-order factors (Markon, 2019). While general intelligence is an important and theoretically valid construct, different analytical approaches should be considered to provide a variety of perspectives on the underlying cognitive subdomains as well (Decker et al., 2020). Since the WISC-V provides a scoring framework for specific index score comparisons, researchers and clinicians must be certain of how well the WISC-V primary indexes represent unique cognitive domains. According to a large body of empirical evidence, bifactor models are often emphasized to be the better choice when it comes to determining and interpreting the relative contribution of each single WISC-V index score independent of *g* (Murray and Johnson, 2013). However, Decker et al. (2020) also pointed out that studies on structural validity using bifactor models or SL procedures might be at least methodologically biased in favor of a presumed and dominant general factor of intelligence. Clinical recommendations for cognitive test interpretation should at least consider these methodological limitations.

In summary, the second-order five-factor model structure that is provided in the WISC-V test manual could not be fully supported by the present EFA and CFA on the extended German WISC-V standardization sample. The absence of two salient indicators for FR in EFA along with slightly worse fit indexes in CFA indicated that dividing VS and FR into distinct factors could be considered inadequate due to over-factoring. Since it could also be shown that only small proportions of true score variance were explained by the first-order factors, interpreting single index scores beyond the FSIQ could likely result in over-interpreting or misinterpreting the true levels of specific cognitive abilities. Consequently, researchers and clinicians should be cautious when interpreting the WISC-V primary index scores individually. Regardless of whether a four- or five-factor structure is considered for the German WISC-V, group factors reflecting the primary indexes do not account for a sufficient proportion of variance to warrant confident interpretation of single index scores. Although the present findings clearly indicate that different levels of specific intellectual domains are not adequately represented by their corresponding WISC-V primary index scores, it has to be noted, however, that the WISC-V was originally designed to measure multiple dimensions underlying a general factor of intelligence. Since Decker et al. (2020) do not recommend to reduce diagnostic decision making to a single test score, clinical case reports should thus include comprehensive analyses that might be at least supplemented by additional clinical information derived from the WISC-V primary indexes. Instead of over-interpreting each single WISC-V primary index score as a valid composite score, these index scores should rather be regarded as pseudocomposite scores representing specific combinations of the underlying primary subtests. If necessary, comparing those pseudocomposite scores as well as performances on the subtest level relative to each other may at best provide additional information about specific cognitive strengths and weaknesses within an individual.

Finally, it has to be noted that the present study only focused on the structural validity of the German WISC-V but did not clarify further questions pertaining to construct validity. Even though Pauls et al. (2019) already examined measurement invariance across gender, for instance, structural validity of the German WISC-V has neither been examined in clinical samples nor in groups with extreme levels of intelligence. Since profoundly gifted individuals are not sufficiently represented in the standardization sample of the German WISC-V, for example, they could feature meaningful cognitive patterns different from those previously described. Therefore, future research should focus on testing the validity of the German WISC-V factor structure on a variety of clinical samples. In order to solve the overall validity disagreements regarding whether hierarchical or bifactor models best describe the structure of intellectual abilities, however, research should go beyond the mere comparison of statistical measures in favor of more theoretical approaches providing an explanatory basis for cognitive constructs in general (Keith and Reynolds, 2018). Research focusing on the integration of psychometrically sound models with neurocognitive outcomes based on brain networks could thus enhance the understanding of the complex nature of human intelligence.

## Data Availability Statement

All rights for the raw data are reserved by the publisher of the WISC-V. Other data supporting the conclusions of this article will be made available by the authors with prior permission.

## Ethics Statement

Ethical review and approval were granted by the responsible school authorities and province school boards in accordance with the local legislation and institutional requirements. Written informed consent to participate in this study was provided by the participants’ legal guardian/next of kin.

## Author Contributions

FP has contributed to data collection, data analysis and interpretation, manuscript preparation, and drafting of the article. MD has contributed to the conception and design of work, data collection, and critical revision of the manuscript. All authors contributed to the article and approved the submitted version.

## Funding

The study was conducted without any funding. The open access publication fees were funded by the library of the Helmut-Schmidt-University/University of the Federal Armed Forces Hamburg.

## Conflict of Interest

The authors declare that the research was conducted in the absence of any commercial or financial relationships that could be construed as a potential conflict of interest.

## Publisher’s Note

All claims expressed in this article are solely those of the authors and do not necessarily represent those of their affiliated organizations, or those of the publisher, the editors and the reviewers. Any product that may be evaluated in this article, or claim that may be made by its manufacturer, is not guaranteed or endorsed by the publisher.
